# Performance of an occupational therapist in a neonatal intensive care unit

**DOI:** 10.25100/cm.v50i1.2600

**Published:** 2019-03-30

**Authors:** María Helena Rubio-Grillo

**Affiliations:** 1 Universidad del Valle, Facultad de Salud, Escuela de Rehabilitación Humana, Grupo SINERGIA, Cali, Colombia

**Keywords:** Occupational therapy, neonatal intensive care, child development, family, infant, premature, extremely premature., Terapia Ocupacional, Cuidado intensivo neonatal, Desarrollo infantil, Familia, infante, prematuro, extremadamente prematuro

## Abstract

**Background:**

The following article constitutes an effort to make explicit an experience in neonatology within the framework of the exercise of occupational therapy, a discipline belonging to the health sciences. The occupational therapist (OT) in the Neonatal Intensive Care Unit in which he participates in an interdisciplinary health group. Exalts the interaction of person-environment-occupation-performance. Encourage self-regulation of the baby. Encourages family participation in co-participation in routine activities.

**Objective:**

To determine the realities and knowledge about the practice of OT in the Neonatal Intensive Care Unit (NICU) by the occupational therapist in the interaction between the baby, the occupation, the caregivers and the environment of the NICU.

**Methods:**

A systematic exploratory review of the performance of the OT in the NICU was made.

**Results:**

The results transcended the thematic variables, the theories, the methods, the approaches, the characteristics of the baby, the occupations, and the contexts of the management of the premature baby.

**Conclusion:**

The education in concepts concerning the occupation of the baby, the interaction with her/his environment and her/his caregivers, the procedures, the guide for the stimulation as the modification of the physical, temporal and social environment facilitate the self-regulation of the baby and we will all be working in pro of your recovery.

## Resumen

**Antecedentes:**

El siguiente artículo constituye un esfuerzo por explicitar una experiencia en neonatología en el marco del ejercicio de la terapia ocupacional, disciplina concerniente a las ciencias de la salud. El terapeuta ocupacional (TO) en la Unidad de Cuidado Intensivo Neonatal como parte del grupo interdisciplinario de salud exalta la interacción de persona-ambiente-ocupación-desempeño. Fomenta la autorregulación del bebé. Alienta la participación de la familia en la coparticipación en actividades rutinarias.

**Objetivo:**

Determinar las realidades y conocimientos sobre la práctica del TO en la Unidad de Cuidado Intensivo Neonatal (UCIN) del terapeuta ocupacional en la interacción entre el bebé, la ocupación, los cuidadores y el entorno de la UCIN.

**Métodos:**

Se hizo una revisión exploratoria sistemática del desempeño del terapeuta ocupacional en la UCIN.

**Resultados:**

Los resultados trascendieron las variables temáticas, las teorías, los métodos, los enfoques, las características del bebé, las ocupaciones, los contextos de la gestión del bebé prematuro.

**Conclusión:**

La educación de conceptos concernientes a la ocupación del bebé, la interacción con su ambiente y sus cuidadores, los procedimientos, la guía para la estimulación como la modificación del entorno físico, temporal y social facilitan la autorregulación del bebé y todos estaremos trabajando en pro de su recuperación.

**Remark**

The following article constitutes an effort to make explicit an experience in neonatology within the framework of the exercise of occupational therapy, a discipline belonging to the health sciences. An occupational therapist in a Neonatal Intensive Care Unit with an ecological practice approach participates in an interdisciplinary health group. The therapist enhances the person-environment-occupation-performance interaction, encourages the baby’s self-regulation and family participation in co-participation in routine activities. The article aims to reflect on the trajectory of the role of the occupational therapist in the interaction between the baby, the occupation, the caregivers, and the neonatal intensive care unit environment. A systematic exploratory review of the performance of occupational therapists in neonatal intensive care units was carried out. The results transcended thematic variables, theories, methods, approaches, characteristics of the baby, occupations, and contexts of the management of premature babies. Finally, it is concluded that in order to facilitate the baby’s self-regulation and teamwork aimed at the child’s recovery it is necessary to be able to access education related to concepts concerning the occupation of the baby, the interaction with his/her environment and his/her caregivers, the procedures, the guide for the stimulation as the modification of the physical, temporal and social environment.

## Introduction

The World Health Organization ^1^ Guidelines stipulates that the assistance given to premature babies who are at risk due to their health conditions need of follow-up sessions guided by the application of theories, approaches, techniques, and methods. One of the most remarkable is the *Sinactiva* theory ^2^, which considers that all human beings have a rich interior life that must be supported in order to develop and prosper otherwise it will be frustrated due to the lack of care, opportunities and the environment surrounding them. The Neonatal Individualized Developmental Care and Assessment Program (NIDCAP) ^3^ ^-^ ^5^ is derived from this theory, as well as the development centered care ^6^, the family centered care ^7^, and it is based on the follow-up and control of premature babies and their families, and the process of acquiring abilities for life. The approaches focused on neurodevelopment ^8^ and sensory integration ^9^ strengthen and dynamize the effects of interventions in the area of health. 

Due to the variety of concepts and approaches in which the practice of health professionals of the neonatal intensive care unit (NICU) is based, in order to determine what the work of the occupational therapist (OT) should be, an exploratory revision was perform which facilitated the knowledge of recent ideas on what has been done, how it has been done, allowing us formulating hypothesis and identifying those aspects that were studied with much detail to identify key concepts, theories, and sources of evidence, such as the type of questions, what strengths and challenges we face in our work and the inclusion of concepts from teams that intervene in the NICU ^10^.

## Materials and Methods

The contents of the bibliographical references found was analyzed, starting with validated and reproducible inferences that could be applied to the performance of the OT in the context of the NICU. The topic was divided into units in order to categorize and interpret the data as it was. Such data as conceptual references, processes, history, tools and strategies was analyzed and in so doing, we could highline and describe some particularities in order to reformulate and generate a complex corpus of knowledge with a harmonic sense for the professional community that works in this clinical area of the profession. 

The following were the biomedical data bases used: Cochrane Library, LILACS, IBECS, EMBASE, ACCESS MEDICINE, MEDLINE (Medical Literature Analysis and Retrieval System Online), SciELO (Scientific Electronic library Online), Redalyc, PUBMED, Scopus, Dial net, HHS Public Access, Sciencedirect. Original articles were included whose content complied with the objective: to determine the realities and knowledge on the practice of the occupational therapist (OT) in the NICU and the interaction between the baby, the occupation, the caregivers and the environment of the NICU where realities and knowledge on the practice of occupational therapy in the NICU will be formulated.

The key words: MeSH, infant, premature, diseases, premature birth, extremely premature, and DeCS defined the verification in the describers of the health sciences: neonatology, occupational therapy in neonatology, the role of the occupational therapist in the NICU, the development centered care and the occupational therapy, the family centered care and the OT. Also included were magazines focused on the performance of the occupational therapist, such as: The American Association of Occupational Therapy, and those from the associations of Australia, Galicia, Chile, Argentina, Great Britain, Canada, and Japan which publish the majority of articles about the profession. We also included magazines on pediatrics and neonatology that provided basic knowledge on the interaction with occupational therapy. 

## Health professionals that assist premature infants at the neonatal intensive care unit

### Conceptual references

Developing the bases for the professional knowledge (researching to know what to do in the practice as therapists) is an exhaustive scrutiny of what it is known and how that knowledge about sample for study was acquired, the manner in which we could validate that knowledge through evidence. It is the building of a careful process, well elaborated, in which the parameters on what it is done that have been more or less agreed upon are complied with. It is applying instruments with standardized guidelines and clinical interventions on children, their parents and the professionals who intervene to measure their efficiency and identify what situations need correction ^11^.

In order to know the sample for the study, it was found that there is a group of babies who due to an illness or a situation present disadvantages impeding their complete participation in their context, according to the National Institute for Health and Care Excellence ^12^. They are premature ^1^ and born too soon ^13^. Currently the newborns of risky pregnancies survive more frequently as a result of advances in obstetric, neonatal and technological care ^14^. These newborns at risk demonstrate immaturity in different corporal structures and in their functions, in the areas of occupational performance, the development of abilities, in the occupational activity in interaction with the environment-context affecting their personal and social participation and it could become a barrier for performing activities that are purposeful and meaningful for daily life ^15^ ^,^ ^16^. 

## Performing careful processes

Due to the infant’s fragility, the vulnerability of their families, the complexity of the medical and social factors that affect them, the health professionals dedicated to the care of newborns at risk guided by world organizations, neonatology associations ^13^ assist those babies with an interdisciplinary or transdisciplinary focus ^17^ ^,^ ^18^ in which each professional guided by their profession own proper standards provide an assistance with expertise in a specialized, timely, individualized and flexible manner, based on knowledge, advanced capacities and abilities minimizing risks or developmental disorders.

Knowing their interventions and therapies, but most of all, knowing the subjects and the group receiving that intervention, it is more evident the contributions made by the interdisciplinary approach. If therapists maintain only a reduced vision of their own disciplinary approach, they assume that these newborns have a physical or health condition resulting from natural causes and won’t conceive the intervention for this population as bio-psychologic-cultural beings. This means that it won’t transcend the interpretation of the occupational realities through the critical analysis offering different theoretic possibilities of interpretation for the occupational phenomena ^9^.

The occupational therapy professionals have a unique set of abilities to contribute to the flexibility and integrity in the management of the premature infant ^19^ ^,^ ^20^. Based on the educational standards that improve the international professional profile proposed by the World Federation of Occupational Therapy ^21^ (WFOT) and the American Occupational Therapy Association (AOTA) ^22^, and the conceptual practical orientation on the functions of the occupational therapist in neonatology where the development of intervention of OT in the NICU is explained ^19^ ^,^ ^20^, besides the different occupations involved in the NICU, in addition to the guidelines proposed for the intervention ^23^ these educational standards promote the education and understanding ^24^, and finally, the requirements of knowledge and the abilities for the practice ^25^.

To be precise about the practices, the proposal made by Kinney ^26^ is revised. Such proposal starts with the concept of the co-participation managed in the interdisciplinary proposal and in the formulation of the Sinactiva ^27^ theory which increases the knowledge on the most vulnerable newborns and their families and makes sure that the opportunities they have would allow them accomplish their potential in order to lead a satisfactory life.

According to commentaries made by Maltese ^2^, Dunn *et al*. ^28^, the theory of Synactiva teaches an individualized approach, it describes the newborn as a being having the capacity to organize and control his/her conduct. It points out that “the child learns about him/herself in his/her environment and finds the way to satisfy his/her needs”. The premature infant who is developing in an unusual environment and who has had less time for the neuro-development in a controlled environment is vulnerable to the sensory stimuli of continuous exposure because they are not neurologically ready to receive an overdose of sensory stimuli. Table 1 exposes the elements of the newborn conduct in the NICU.

**Table 1:** Newborn conduct in the NICU

Newborn conduct in the NICU	
Motor	Measures muscular tone, movement, activity, and posture
Autonomous	Measures skin color, cardiac frequency, and respiratory pattern
States of consciousness	Categorizes the level of the central nervous system as far as awareness, sleep, awakening, cry, demonstrating the strength and modulation of his/her states and patterns of transition between each of them.
Attention interaction	Capacity to interact with the environment.
Self-regulation	Measures efforts to get balance using other subsystems.

Source: Vergara *et al* ^29^ ^).^

 On the other hand, Als ^27^, pioneer of the assistance during development, expresses that the intrauterine sensorial experience is dangerous for premature infants for the neurological structure of the immature brain. Based on the concepts presented in the Synactiva theory, it was created the approach for the assistance in the NICU on part of the RN and its individualized development called “development care”. For this reason, the evaluation program NIDCAP ^4^ is focused in improving the comprehension of the caregiver in self-regulated capacities for the infant is based in observing the baby’s conduct as communication in itself. This program is centered in the skills and strengths of the individual infants. It is focused in the integration and modulation of the sensory system and supports all the infant’s care givers: parents and professionals. This program takes into account the autonomous motor system, the states of awareness, attention and interaction, and the self-regulation as it is described in Table 1. (The definition of each one of them).

Starting from the synthesis and the growing of the Synactiva theory, concepts that have been recognized as approaches in the NICU are consolidated. Such concepts are: NIDCAP, development centered care, family centered care, neurodevelopment and sensory integration. Each one of these contains compatible elements, they have the same bases, methods and tools and they are interrelated. The analogy between the Sinactiva theory and the intervention of OT are described in Table 2.

**Table 2:** Analogy relating the Occupational Therapy with the theory of Synactiva

Infant’s activities	Activities with sensory visual components: audio, oral, tactile, proprioceptive, vestibular. Daily activities of feeding, sleep, and rest
Infant’s factors	Self-regulation
	Tone, strength, resistance, posture control, oral control
	State of awareness, modulation, transition of states
	Visual, auditive, and developmental skills
Contextual factors	Physical context**:** level of activity, connection, modulation
	Social context - Cultural (caregiver). sensibility, compromise, willingness, number
	Temporal context: rhythm, routine, medical needs

Source: Als^27^

The NIDCAP ^3^ ^-^ ^5^ derived from the Sinactiva theory, starts with observation of the infant’s response to the care procedures. Based on this, individualized recommendations and strategies are contributed for his/her care. These recommendations support the physiological stability of the infant as well as his/her self-regulation and the organization of his/her behavior Compatible with the NIDCAP, the OT centers its performance in the interaction of the autonomous, motor systems, the organizational state, the attention interaction and the regulatory subsystems among themselves and with the environment context according with the ecology and the human performance ^28^ ^,^ ^29^.

The following are the approaches being taken into consideration: the developmental care ^30^ ^,^ ^31^, similar with the performance of the OT, it favors the process of neurodevelopment and its main characteristic is the individualization of the RN care starting from observation of their conducts, the propagation of a standardized instruction method, the availability of an environment that supports the participation of the newborn and his/her family in the expected activities which favors the reduction of environmental stress factors as well as improving the micro-environment (light, sound, movement, position, tact, calmness, and physical support) to maintain an optimal tone and a quiet and refreshing sleep in a relaxed, comfortable or vigilant environment ^32^ ^-^ ^37^.

Another approach is that of family centered care ^38^ ^-^ ^43^. It is also compatible and similar, since with this approach the actions in the macro-environment are shared. The intervention rotates around the parents so they can recognize their children behaviors and get integrated in their care ruled by the principles of closeness to the family and participation of the newborn care and on the other hand, around the professionals in the participation in interdisciplinary work and in the change of attitudes, in the processes and in each profession own dominium ^44^.

The next approach is that of neurodevelopment ^8^ ^,^ ^30^ ^,^ ^32^. It legitimates the fact that a stable and adequate infant posture favors the correct perception and a variety of stimulus for the future planification and motor coordination. The OT shares this approach with other rehabilitation professionals in order to reach self-regulation and organization in the transition of movement, the posture alignment that provide the entry to sensory stimuli and help the infant mature at the motor, perceptive, and sensorial levels ^45^.

As part of the therapeutic tool, the occupational therapist uses the sensory integration approach ^9^ ^,^ ^46^ ^-^ ^48^. He/she assumes that the capacity of the infant to process the sensory impulse and integrate it with another sensory information serves him/her to use that capacity for adaptive purposes to improve his/her skills of performance, if and only if the environment would offer him/her the type or the adequate quantity of sensory experiences ^49^.

As it is evident in the complex environment of the NICU, the intrinsic importance of the specialized medical assistance is highlighted as well as the hyper-technical and the interaction of inter and multidisciplinary teams. The team’s participation is based on the concepts of individualized assistance centered in the family and they take responsibility for coordinating the practice evaluation to comply with the parameters of developmentally oriented assistance, insisting in having the appropriate environment for the infant and his/her family ^17^ ^,^ ^18^.

The OT as part of the interdisciplinary team establishes identity in the discipline (it does not have to be exclusive or excluding) and it differs from others in that the popular imaginary tends to be materialized with occupational therapy. It bases its interventions on concepts and practices defined by the WFOT ^50^ and the AOTA ^51^ that establish that “the profession is centered in empowering and facilitate the participation of persons or groups in taking roles, habits, and routines at home, at school, at the workplace, and at the community through the occupation. It centers the therapeutic use of activities of the daily life (occupations) with individuals or groups in order to empower or facilitate the participation”. This is coherent with the principles of the international classification of functioning and handicap (ICF) ^52^ mentioned by the WHO, but also in situations closer to neonatology, based on the model of the person, occupation, and environment (POE) ^53^ ^,^ ^54^ in which attention to the nature of occupational performance is given starting from the dynamic and interactive relation of the person (infant, family) with the components and the areas of yielding (co-occupation, adaptation, communication skills) and the environment as a critical factor in the human performance (physical, social, cultural, and temporary) that influences the behavior and the occupation ^55^. 

## The historical roots found the development of knowledge, attitudes, and practices of the being, doing and feeling of the occupational therapy 

The professional performance of the OT in the NICU has leaded a trail through time and space. This allows having an understanding of the conceptual and practical configuration, consolidating the performance of the OT in neonatology, recognizing the infant’s development, his/her occupation in the context in which he/she is developing, the interaction with the family, identifying the contributions made by interdisciplinary groups in the research, and registering the influence in the social and labor contexts.

Taking into account the historical trail, Gorga ^56^ taught the developments in the occupational therapy practice for hospitalized children in a NICU. She mentions the representatives of each event or epoch. In her writings, it is summarized the beginning of how the approaches are applied in the treatment for medically stable infants with developmental handicaps and their families in the intensive care unit environment.

During the 1980s, it came to be known specifically the beginning of the application of the Sinactiva theory. It was when practices were beginning to be modified according to the guidelines of development to examine the effects of manipulation on children and the therapy could be integrated to the plan of nursing care. It was specified if the benefits overcame the risks, if the changes could be measured and if they contributed to the results. Vergara *et al*. ^19^ ^,^ ^20^, recommended that the therapy were centered on nursing infants and oriented to the parents to whom it was taught the specific skills such as feeding, and an easy transition from the NICU to the home environment was facilitated.

At the end of the 1980s and the beginning of the 1990s, the paring of therapists and parents was established as a movement of collaborative interchange or society. It was specified that as long as the concepts of collaboration, communication, and empowerment were applied, they could guide the way for the near future. Also Schfsky ^57^, Cui *et al*. ^58^, and Ross *et al*. ^59^, define the guidelines that a neonatal therapist requires as part of the personnel of the NICU at an assistance III or IV levels that includes assistance to infants at any gestational age at birth with medical and complex surgical needs. They define the roles of the OT in the NICU and the use of different therapeutic interventions performed on nursing infants at high risk at the NICU. They express that due to the vulnerability of the premature infants, the therapist based in the NICU need advanced skills to optimize the infant’s results at the same time that they understand and are adapted to the medical interventions that occur simultaneously with the therapeutic ones.

## Tools for the intervention of the occupational therapist facilitate the interpretation of their usage

Up until now it has been presented the revision about the approaches, methods, and theories that the occupational therapist uses in the NICU as tools for intervention. Starting from this intervention it is understood that the movement, organization of the muscular tone, the activity and posture help the infant’s self-regulation. The effort the infant makes to get balance in the organization activities as well as the attention-interaction manifests his/her capacity to interact with the environment. The neuro-regulatory capacity of each child guides the interventions geared towards optimizing the results of the neurological development ^20^ ^,^ ^28^.

The OT in neonatology, a participant of the interdisciplinary group ^17^ ^,^ ^18^, is guided, as it was already announced, by the standards determined by the WHO ^1^, AOTA ^60^, and those proposed by Adrados ^61^ and Vergara *et al* ^62^. He/she employs specific approaches treating the infant as a singular being that acts with physical, cognitive, affective, and individual factors manifested in a range of abilities that consciously interact with the temporal, social, cultural, and physical environment in which the infant develops. Such factors are described in Table 3.

**Table 3:** Inter-related factors in the occupational performance of the newborn

DCC (development centered care)	Immediate surroundings
	Moment, sequence, and adaptation during care
	Support for self-regulation during rest
	Support for self-regulation during care
	Appropriate sensory experiences according to development
FCC (family centered care)	Unit environment
	Communication
	Participation in care
Neurodevelopment	Provides sensory-motor experiences
	Promotes the development of normal movements
	Development of postural patterns

Source: Vergara *et al* ^20^

According to what is represented, the OT recognizes the infant as an occupational being who performs activities, is an active co-participant of action patterns that emerges from the interaction of the child with the environment. Kathleen ^63^ considers these occupations as “the tasks and activities that are valued whether in the family culture or in the NICU, and in which it is expected the child to participate”. “The occupations are activity patterns that emerge from the interaction of the child with the environment”. This way, it could be established that the occupational performance is the occupation of the infant in tasks and activities that are expected whether by the family or by the NICU personnel and in which there is an idea that becomes synchronic between the co-occupational experiences of the infant and those of the caregivers. This thought induces the occupational therapist to couple the newborn nature with adaptations and modifications in the infant, the caregiver in the environment or the objects inside the NICU ^64^ ^-^ ^66^. 

Then, the OT uses a transactional relation between the newborn, his/her activities, his/her valued occupations and his/her context and manages therapeutically the daily life activities because he or she observes the state of organization with which interacts as Hunter *et al*. ^23^, explains. The OT empowers or facilitates the participation of the caregivers in the creation of habits and routines and the sensory interactions as well as the modulations of sensations, of the space and objects as tools ^67^ ^,^ ^68^.

It is for the above mentioned that the role of the OT in the neonatology services is geared towards the intervention and research on the acquisition and preservation of abilities of those newborns that have or are at risk of developing a disease, a lesson, a deficiency, a handicap, a limitation of activity or restriction from participation, involving the relatives ^68^ ^-^ ^70^. 

Retaking the systematic revision of the articles aiming towards the actualization of contents and concepts, it was found that the OT interventions in neonatology are not rules nor prescriptions, they are individualized specific actions for each child at every moment. Part of the observation of the infant response to the changes or suggestions is having the responsibility, firs and foremost, of preserving the neuro-behavioral and physiological stability actively involving the family to the health care team. This is done with the objective to influence the present and future quality of life of the child and of his/her family as they face situations they most confront ^71^ ^-^ ^74^. 

## Methodological strategies and techniques used by the ot in his/her occupational performance 

Here an approximation is made to the fact that strategies and technics of recollection and production of data are working around the nature of the premature infants. From the observations made to describe data from the talks or interviews done to determine an occupational profile, it is aimed at the meaning the professionals give to health, the standardized guidelines to measure the occupational performance of the newborn and his/her family, and the techniques used in their interventions and research ^75^ ^,^ ^76^.

Since many newborns are examine by professionals knowledgeable about infant development, those who are involved in the diagnosis and interventions should recognize that besides the medications, there are other approaches proposed by other disciplines such as OT. They propose games and ludic activities as ways of stimulating the senses and promoting social skills, movement control, and cognitive development. To that end, it is important to document the intervention and impact this have on the infant and his/her family ^71^ ^-^ ^76^ routine daily life.

In order to develop his/her practice in the NICU, the OT needs to know about the conditions, medical procedures, and vulnerabilities of the newborn. In addition, he/she will recognize an understanding of the individualized developmental skills, the competencies of the neonatal ^19^ ^,^ ^20^ neuro-behavioral organization theories, the family life, the principles of socio-emotional development, and the team work, among others. This is the only way in which the OT could comprehend how these factors interact to influence the newborn performance. According to what is mentioned, the thinking, doing, and being of the OT in neonatology ranges from the secondary prevention through the detection to the diagnosis. Among these components of action there is knowing the believes, the cultural practices, health needs, occupational opportunities, political systems, ideologies and images behind the medical or clinical interventions ^76^.

During the evaluation / intervention, the OT keeps in mind the neuro-behavioral organization, the sensorial development and process ^77^. On the other hand, he/she considers the refer motor function at a posture level respecting the flexor pattern of arms and legs moving together to the center of the body, giving the characteristic fetal position in a symmetrical manner or corporal mechanics. The OT uses nests and rolls for comfort, contention, extreme stability, favoring the flexor posture and avoiding cranial deformities. Similarly, he/she takes into account the daily activities (feeding, clothing, changing dippers, transferring, transition states, sleeping) as well as the socio-emotional development ^78^ ^-^ ^80^.

As far as the family is concerned, its systems continue, thinking on the learning styles for adults, expectancy, cultural growth expectancy, parent-children interactions as well as the role of the father during the hospitalization, the family centered informed care and what it is more important, the transition of the infant from the hospital to his/her home ^81^.

A key aspect is the environment ^82^ ^-^ ^84^.

In the sensorial environment five types are considered:

- Tactile; synchronization, intensity, texture, management for procedures, and interaction with parents. The tactile proprioceptive gives a facilitating impulse for self-organization.
- Proprioceptive-vestibular: synchronization, intensity, texture, management for procedures, and interaction with parents. (hammocks, balance of the infant in his/her mother’s lap)
- Specific olfactive, taste experiences in the NICU (time, quality, and intensity)
- Auditive: intensity, duration, time, animated Vs. inanimate
- Visual: Temporization, environment and intensity of focal light, content and visual field.

Social environment in which parents and infants, extended family members and babies, personnel and babies; parents and personnel; occupational therapist, parents, personnel and infants are identified. 

The physical environment is formed by the teams and medical procedures; the frequency, time, duration, quality and intensity of sensory information of doctors, team and procedures; the sensory information of the teams, procedures and personnel activities that are harmful for the neuro-behavioral organization.

The cultural environment refers to the philosophy of specific care in the NICU, including its particular orientation towards the acute and chronic care of the infants; the roles, functions, attitudes, and the position of the team members in the structural organization of the individual NICU; the influence of stressful factors of the NICU, the communication and structure patterns, both formal and informal among the personnel members and between the family and the personnel; rules of conduct explicit and non-explicit; the effect of the physical and social environment in the personnel performance and moral and the humanization of the NICU (incubator with personal elements and family souvenirs).

So being, one integral part of the process of occupational therapy is the therapeutic use of oneself and this makes possible to develop and manage his/her therapeutic relation through the use of the narrative and the clinical reasoning (empathy and a collaborative approach). Through the use of interpersonal communication techniques, the power of the relation is displaced so that the families would have a greater control in the decision making and problem solving. These are essential aspects for an efficient intervention ^83^ ^,^ ^84^. 

Summarizing, in order to identify the treatment interventions used inside the NICU by the OT, an example of Sardá was taken. He presented some forms of making interventions with newborns at high risk that could be a guide for the approach. Table 4 announces the techniques for the discussion of environmental and unimodal sensory stimulation ^85^. 

**Table 4:** The techniques: reduction of environmental stimulation. Sensory unimodal stimulation

Reactions to assistance/interaction	
Facing signs of stress, suspend intervention. One sensory stimulation at a time. Soft lights	Keeping intervention when facing signs of stress. Interaction under inadequate conditions. More than 1 sensory stimuli at a time.
Keep the infant covered or dressed. Visual stimuli at 30-40 cm from sight. Eliminate unnecessary stimuli. Facilitate hand-mouth or pacifier activity. Posture in flexion. Give support and stability. Intervention RN in awareness.	

Source: Sardá ^85^ ^).^

## Conclusions

The previously exposed represents the general vision of the requirements for the OT approach in neonatology.

Regarding the development of premature infants, it is considered that if the individual needs are not stimulated in a long run, the damages will be evident. Such damages could be: the capacity for praxis, imitation, sequencing, and construction; the motor skills, the cognitive capacities, such as perception; processing abilities (capacity of organizing actions in an opportune and safe manner) and the capacities of emotional regulation that could affect the ability to efficiently respond to the demands of occupation with a range of emotions.

Consequently, beyond the reference model centered in the conventional problem of handicap, the OT make a valuable contribution to the newborn care because they center in the power of commitment with the occupation and to the support of the parents in the infant’s care in order to accomplish positive results for the family and the caregivers.

It is a change of attitude from **“we are the experts and we know what is best for the patient”** to **“we will do it with you”** Zarubi *et al* ^86^.

If we understand the interdisciplinary concept as a dialogue and interchange of techniques, concepts and researching methods among different disciplines, the OT, without losing his/her identity, does not have a problem in grasping the heuristic tools from other disciplinary fields for the research work. The intervention of the OT in the NICU is a collaborative practice in which it is demonstrated at the same time respect and support to the functions of other people and the disciplines in the lives of the newborns.

Knowing the nature of the social, cultural structure, believes, cultural practices, health needs of relatives and health professionals provides occupational opportunities relevant to the interactions of the OT that are behind the medical or clinical interventions.

Sharing our experiences, successes and errors, difficulties and wrongs, generating and validating instruments, standardized guidelines and clinical interventions, measuring its efficacy and identifying situations to be corrected presents us as “active researchers” that teach not only what has been done, but also what is been doing and what can change. Counseling and orientations based on real research experiences could contribute to the “how” to face to the process of research starting from the documented observation, the interventions, its impact in propagating and publicly communicating. This way, we are building history and practices of the OT in neonatology is a symbol for formulating the practical and transforming character of the occupational praxis.
