# Supplementary material for: Performance of an occupational therapist in a neonatal intensive care unit
Source: Colomb Med (Cali). 2019 Mar 30;50(1):30–9. doi: 10.25100/cm.v50i1.2600 (PMC6536043; doi:10.25100/cm.v50i1.2600)
Supplement: Related texts

# Review: Desempeño profesional del terapeuta ocupacional en la Unidad de Cuidado Intensivo Neonatal

**Comentarios**

Este artículo constituye un esfuerzo para hacer explícita una experiencia en neonatología en el marco del ejercicio de la terapia ocupacional, una disciplina que pertenece a las ciencias de la salud. Un terapeuta ocupacional en una unidad de cuidados intensivos neonatales con un enfoque de práctica ecológica participa en un grupo de salud interdisciplinario. El terapeuta mejora la interacción persona-ambiente-ocupación-desempeño, fomenta la autorregulación del bebé y la participación de la familia en la coparticipación en las actividades de rutina. El artículo pretende reflexionar sobre el papel del terapeuta ocupacional en la interacción entre el bebé, la ocupación, los cuidadores y el entorno de la unidad de cuidados intensivos neonatales. Se realizó una revisión exploratoria sistemática del desempeño de los terapeutas ocupacionales en unidades de cuidados intensivos neonatales. Los resultados trascendieron variables temáticas, teorías, métodos, enfoques, características del bebé, ocupaciones y contextos del manejo de bebés prematuros. Finalmente, se concluye que para facilitar la autorregulación y el trabajo en equipo del bebé para la recuperación del niño es necesario poder acceder a la educación relacionada con los conceptos relacionados con la ocupación del bebé, la interacción con su entorno y su entorno. Sus cuidadores, los procedimientos, la guía para la estimulación como la modificación del entorno físico, temporal y social.

## Introducción

Los lineamientos de la Organización Mundial de la Salud (OMS) ^1^ la atención de los bebés prematuros en riesgo por sus condiciones de salud, tienen un seguimiento guiado por la aplicación de teorías, enfoques, técnicas, métodos. Entre ellos, sobresale la teoría Sinactiva ^2^, que considera que todos los seres humanos tienen una vida interior rica que debe ser apoyada para desarrollarse y prosperar o se verá frustrada por el cuidado, las oportunidades y los entornos que se encuentran. De ella se deriva el *Neonatal Individualized Developmental Care and Assessment Program* (NIDCAP) ^3^^-^^5^, Cuidado Centrado en el Desarrollo (CCD) ^6^, Cuidado Centrado en la Familia (CCF) ^7^, y se fundamentan en el seguimiento y control de los prematuros y sus familias, el proceso de adquisición de habilidades para la vida. Los acercamientos de neurodesarrollo ^8^ e integración sensorial ^9^ fortalecen y dinamizan el efecto de las intervenciones en el área de salud.

Debido a la variedad de conceptos y acercamientos que fundamentan la práctica de los profesionales de salud en Unidad de Cuidado Intensivo Neonatal (UCIN), para determinar cuál es el desempeño del Terapeuta Ocupacional (TO) se realizó una revisión exploratoria, que facilitó conocer las ideas actuales, lo que se ha hecho, de qué manera, lo que permitió generar hipótesis e identificar aquellos aspectos que fueron estudiados con más detalle para identificar conceptos clave, teorías y fuentes de evidencia, como los tipos de preguntas las fortalezas y desafíos de la experiencia en nuestro trabajo y la inclusión de conceptos de equipos que intervienen en la UCIN ^10^. 

## Materiales y Métodos

Los datos bibliográficos encontrados se expusieron a un análisis de contenido a partir de inferencias reproducibles y válidas que podían aplicarse al desempeño del TO en el contexto de la UCIN. El tema se descompuso en unidades para categorizar e interpretar los datos como fueron los referentes conceptuales, procesos, historia, herramientas y estrategias y así destacar y describir particularidades a fin de replantear y generar un complejo cuerpo de conocimientos con un sentido armónico para la comunidad profesional que se desempeña en esta área clínica del ejercicio profesional.

Las bases de datos utilizadas fueron las biomédicas: Cochrane Library, LILACS, IBECS, EMBASE, ACCESS MEDICINE, MEDLINE (Medical Literature Analysis and Retrieval System Online), SciELO (Scientific Electronic library Online), Redalyc, PUBMED, Scopus, Dial net, HHS Public Access, Sciencedirect. Se incluyeron los artículos originales, cuyo contenido respondiera al objetivo: Determinar las realidades y conocimientos sobre la práctica del TO en la UCIN del terapeuta ocupacional en la interacción entre el bebé, la ocupación, los cuidadores y el entorno de la UCIN donde se plantearan las realidades y conocimientos sobre la práctica del TO en la UCIN.

Las palabras claves definieron la verificación en los descriptores de las ciencias de la salud MeSH: Infant, Premature, Diseases. Premature Birth. Infant, Extremely Premature y DeCS: neonatología; terapia ocupacional en neonatología; el rol del terapeuta ocupacional en la UCIN; cuidado centrado en el desarrollo y la terapia ocupacional; cuidado centrado en la familia y la terapia ocupacional. También, se incluyeron las revistas propias del desempeño del terapeuta ocupacional como son la Asociación Americana de Terapia Ocupacional (AOTA), la Australiana, la Gallega, Chilena, Argentina, Británica, Canadiense, Japonesa, por difundir la mayoría de los artículos de la profesión, además revistas de pediatría, neonatología, las cuales aportaron los conocimientos de base y la interacción con terapia ocupacional. 

## Profesionales de salud que atienden a los prematuros en la Unidad de Cuidado Intensivo Neonatal

## Referentes conceptuales

Desarrollar la base del conocimiento profesional (investigar para saber qué hacer en la práctica como terapeutas) es un exhaustivo escrutinio de lo que se sabe y cómo se sabe acerca del colectivo de estudio, la forma en que podemos validar el conocimiento a través de la evidencia. Es la realización de un proceso cuidadoso, bien elaborado, en donde se cumplen los parámetros más o menos consensuados de lo que se hace; es aplicar instrumentos con pautas estandarizadas e intervenciones clínicas a niños, los padres de los niños y los profesionales que los intervienen, para medir su eficacia e identificar situaciones por corregir ^11^. 

Para conocer el colectivo de estudio se encontró que existe un grupo de bebés que por una afección o situación presenta desventajas frente a la participación plena en su contexto, según el *National Institute for Health and Care Excellence* ^12^, Prematuros ^1^ y Nacidos Demasiado Pronto ^13^. Los recién nacidos (RN) producto de embarazos de riesgo, actualmente sobreviven con mayor frecuencia, como resultado de los avances en los cuidados obstétricos, neonatológicos y tecnológicos ^14^. Esos recién nacidos en riesgo demuestran inmadurez en las diferentes estructuras corporales y su función, en las áreas de desempeño ocupacional, el desarrollo de habilidades; en la ejecución ocupacional en interacción con el ambiente-contexto; lo cual repercute en la participación personal y social y se puede convertir en barreras en la realización de actividades con propósito y significado de la vida diaria ^15^^,^^16^.

## Realización de procesos cuidadosos 

Debido a la fragilidad de los bebés, la vulnerabilidad de las familias, la complejidad de los factores médicos y sociales que los afectan, los profesionales de la salud dedicados al cuidado del RN en riesgo, regidos por las organizaciones mundiales, las asociaciones de neonatología ^13^ atienden desde el enfoque interdisciplinario o transdisciplinario ^17^^,^^18^, donde cada profesional, direccionado por los estándares de práctica propios de su profesión, aporta una atención especializada, experimentada, oportuna, individualizada y flexible, cimentada en conocimientos, competencias y habilidades avanzados, que minimizan los riesgos o los trastornos en el desarrollo.

Al conocer sus intervenciones y terapias, pero, por sobre todo, a los sujetos y colectivos de sus intervenciones, se hacen más evidentes los aportes de lo interdisciplinario. Si los terapeutas solo se quedan con una visión reducida de lo disciplinario, asumen que estos neonatos ostentan una condición física o de salud producto de causas naturales y no conciben la intervención en esta población como seres “biopsicosocioculturales”, es decir, no trasciende a la interpretación de las realidades ocupacionales, a través de los análisis críticos, ofreciendo diferentes posibilidades teóricas de interpretación de los fenómenos ocupacionales ^9^.

Los profesionales de terapia ocupacional poseen un conjunto único de habilidades para contribuir a la flexibilidad e integralidad en el manejo del prematuro ^19^^,^^20^. Afirmados en los estándares educativos que mejoran el perfil internacional de la profesión, propuestos por la Federación Mundial de Terapia Ocupacional ^21^ (FWOT) y la AOTA, y las orientaciones conceptuales, prácticas, funciones del terapeuta ocupacional en neonatología donde se explica el desarrollo de intervencion del (TO) en la (UCIN) ^19^^,^^20^, las diferentes ocupaciones involucradas en la UCIN ^22^, además de las guías propuestas para la intervención ^23^ promueve la educación y entendimiento ^24^, y finalmente los requerimientos del conocimiento y las habilidades para la práctica ^25^.

Para precisar sobre ellas, se repasa la propuesta de Kinney ^26^ que parte el concepto de la coparticipación manejado en la propuesta de interdisciplinariedad y del planteamiento de la teoría Sinactiva ^27^, la cual aumenta el conocimiento y la comprensión de todo el desarrollo humano, para mejorar las vidas de los recién nacidos más vulnerables y sus familias, y asegurar que las oportunidades que tienen les permitan cumplir sus potenciales para llevar una vida satisfactoria. 

La Teoría Synactiva, Maltese ^2^, Dunn *et al* ^28^ comentan, enseña un enfoque individualizado, describe al neonato como un ser con capacidad para organizar y controlar su conducta; señala que “el niño aprende acerca de sí mismo y de su entorno, y encuentra la forma de satisfacer sus necesidades”. El bebé prematuro que se está desarrollando en un entorno inusual y ha tenido menos tiempo para el neurodesarrollo en un entorno controlado es vulnerable a los estímulos sensoriales de exposición continua, porque no están neurológicamente listos para integrarse a la sobrecarga sensorial. En la Tabla 1 se señalan los elementos del comportamiento del neonato en la UCIN.

**Tabla 1:** Comportamiento del neonato en la UCIN.

Comportamiento del neonato en la UCIN	
Motor	Valora el tono muscular, movimiento, actividad y postura
Autonómico	Valora el color de la piel, la frecuencia cardiaca y el patrón respiratorio.
Estados de conciencia	Categoriza el nivel del sistema nervioso central en cuanto a vigilia-sueño-despertar-llanto, demostrando la robustez y modulación de sus estados y patrones de transición de uno a otro.
Atención-interacción	Capacidad para interactuar con el medio.
Autorregulación	Valora los esfuerzos para conseguir el balance con los otros subsistemas

Fuente: Vergara *et al*^29^^).^

Por otro lado, Als ^27^, pionera de la atención del desarrollo, expresa que la experiencia sensorial extrauterina para los bebés prematuros es perjudicial para la estructura neurológica del cerebro inmaduro. Con base en los conceptos presentados en la teoría Synactiva, se creó el enfoque para la atención en la UCIN del RN y su desarrollo individualizado, llamado "Cuidado del desarrollo", por esto, el Programa de evaluación NIDCAP ^4^ se enfoca en mejorar la comprensión del cuidador en capacidades autorregulatorias del bebé, se basa en ver el comportamiento del bebé como comunicación en sí mismo, se centra en las competencias y fortalezas de los bebés individuales, se enfoca en la integración y modulación del sistema sensorial y apoya a todos los cuidadores del bebé, padres y profesionales. Tiene en cuenta el sistema motor, autonómico, los estados de alerta, atención e interacción, y la autorregulación como se describe en la Tabla 1 la definición de cada uno de ellos

A partir de la síntesis y el crecimiento de la teoría Synactiva, se consolida conceptos que han sido reconocidos como enfoques en las UCIN, de NIDCAP, cuidado centrado en el desarrollo, cuidado centrado en la Familia, Neurodesarrollo e Integración Sensorial, cada uno contiene elementos compatibles, tienen la misma base, los métodos y herramientas y se interrelacionan. En la Tabla 2 se describen las analogías entre la teoría Sinactiva y la intervención de TO. 

**Tabla 2:** Relaciones análogas de la terapia ocupacional con la teoría Synactiva.

Ocupación del bebé	Actividades con componente Sensorial Visual. Auditivo, Oral, Tactil, Propiocetivo, Vestibular. Actividades cotidianas de alimentación, sueño, descanso
Factores del bebé	Autorregulación
	Tono, fuerza , resistencia, control postural, control oral
	Estado de alerta, modulación, transición de estados
	Destrezas visuales, auditivas, de desarrollo
Factores contextuales	Contexto físico**:** Nivel de actividad, Contención, Modulación
	Contexto social - Cultural (cuidador). Sensibilidad, Compromiso, Disposición, Numero
	Contexto Temporal: Ritmo, Rutina, Necesidades médicas

Fuente: Als ^27^

El NIDCAP, ^3^^-^^5^ derivado de la teoría Sinactiva, parte de la observación de la respuesta del bebé a los procedimientos de cuidado; con base en ella, se aportan recomendaciones y estrategias individualizadas para el cuidado, que apoyan la estabilidad fisiológica del bebé, la autorregulación y la organización del comportamiento. Compatible con el NIDCAP el TO centra su actuación en la interacción de los sistemas autonómicos, motores, el estado-organizativo, la atención-interacción y los subsistemas reguladores entre sí y con el medio ambiente contenido de acuerdo a la ecología de la ejecución humana ^28^^,^^29^.

Los siguientes son los enfoques tenidos en cuenta: el *Cuidado del Desarrollo* ^30^^,^^31^*;* análogo con la actuación del TO, favorece el proceso de neurodesarrollo y su principal característica es la individualización de los cuidados del RN a partir de la observación de sus conductas, la promulgación de un método de instrucción estandarizado, la provisión de un entorno que apoya la participación del neonato y su familia en las ocupaciones esperadas; lo que favorece la reducción de los factores de estrés ambiental, como mejorar el microambiente (luz, sonido, movimiento, posición, tacto, calma, soporte físico) para mantener el tono óptimo y sueño tranquilo y reparador en un ambiente relajado, cómodo o de vigilia ^32^^-^^37^. 

Otro enfoque es el de *Cuidados Centrados en la Familia,* ^38^^,^^43^ también, hay compatibilidad análoga, ya que con este enfoque se comparten las acciones en el macroambiente. La intervención gira alrededor de los padres para que reconozcan las conductas de sus hijos y se integren en los cuidados regidos por los principios del acercamiento a la familia en la participación en los cuidados del RN y por otro lado en torno a los profesionales en la participación en el trabajo interdisciplinario y el cambio de las actitudes, en los procesos y los dominios de cada profesión ^44^. 

El siguiente es el enfoque de *neurodesarrollo* ^8^^,^^30^^-^^32^ legitima que una postura estable y adecuada del bebé favorece la percepción correcta y variada de estímulos para la futura planificación y coordinación motora. La TO comparte este enfoque con otros profesionales de rehabilitación, con el objetivo de alcanzar la autorregulación y la organización en la transición del movimiento, el alineamiento postural, que provean las entradas sensoriales de estímulos y ayuden al bebé a madurar el nivel motor, perceptivo y sensorial ^45^. 

Como parte de su herramienta terapéutica, el terapeuta ocupacional utiliza el acercamiento de *integración sensorial* ^9^^,^^46^^-^^48^; asume que la capacidad del bebé para procesar el impulso sensorial e integrarlo con otra información sensorial es para usarlo adaptativamente y mejorar así sus capacidades de desempeño; esto siempre y cuando el entorno ofrezca el tipo o la cantidad adecuada de experiencias sensoriales ^49^. 

 Como se evidencia en el entorno complejo de la UCIN se resalta la importancia intrínseca de la atención médica especializada, la hiper-técnica, y la interacción de equipos inter y multidisciplinarios. La participación de los equipos se basa en los conceptos de atención individualizada y centrada en la familia y se responsabilizan de coordinar la evaluación de la práctica, para que cumpla los parámetros de atención orientada al desarrollo, insistiendo en el ambiente apropiado para el bebé y su familia ^17^^,^^18^.

El TO como parte del equipo interdisciplinario establece identidad en la disciplina (no tiene que ser una exclusiva y excluyente) y se diferencia de otras que en el imaginario popular tienden a mimetizarse con la terapia ocupacional. Para su intervención se fundamenta en conceptos y prácticas definidos por WFOT ^50^ y la AOTA ^51^, que establecen que “La profesión se concentra en potenciar o facilitar la participación de personas, grupos, en la realización de los roles, los hábitos y las rutinas en el hogar, la escuela, el lugar de trabajo y la comunidad a través de la ocupación. Centraliza el uso terapéutico de las actividades de la vida cotidiana (ocupaciones), con individuos o grupo, con el fin de potenciar o facilitar la participación”; esto es coherente con los principios de la Clasificación Internacional del Funcionamiento y la discapacidad (CIF) ^52^ mencionados por la OMS. Pero también, en situaciones más afines en neonatología, se fundamenta en el modelo de la persona, la ocupación y el entorno (PEOP) ^53^^,^^54^ en el cual se presta atención a la naturaleza del desempeño ocupacional a partir de la relación dinámica e interactiva de la persona (el bebé, la familia) con los componentes y las áreas de rendimiento (co-ocupación, la adaptación, las habilidades de comunicación), y el ambiente como un factor crítico en la ejecución humana (físico, social y cultural y temporal), que influye en el comportamiento y la ocupación. ^55^

## Las raíces históricas fundamentan el desarrollo del conocimiento actitudes y prácticas del ser, hacer y sentir de la terapia ocupacional

El desempeño profesional del TO en la UCIN ha alcanzado un recorrido a través del tiempo y espacio, lo que permite entender la configuración conceptual y práctica; consolidar la actuación de los TO en neonatología; reconocer el desarrollo del bebé, su ocupación en el contexto donde se desarrolla, la interacción con la familia; identificar los aportes en los grupos interdisciplinarios en la investigación, y registrar la influencia en el contexto laboral y social. 

A propósito del recorrido histórico, Gorga ^56^ ensambla los desarrollos de la práctica de terapia ocupacional para niños hospitalizados en una UCIN. Menciona los representantes de cada evento o época, en los escritos se sintetiza el comenzó de cómo se aplicaron los enfoques en el tratamiento de bebés médicamente estables, con discapacidades del desarrollo y sus familias, en el entorno de cuidados intensivos.

Durante la década de 1980, se dio a conocer específicamente el inicio de la aplicación de la teoría Sinactiva. Fue cuando se empezaron a modificar las prácticas de acuerdo con las pautas de desarrollo, a examinar los efectos de la manipulación de los niños, y la terapia que podría integrarse en el plan de cuidados de enfermería. Se especificó si los beneficios superaban los riesgos, si los cambios eran medibles y si contribuían al resultado. Vergara *et al*. ^19^^,^^20^, recomendó que la terapia fuera centrada en los lactantes y orientada a los padres, a quienes se les enseñaba habilidades específicas, tales como la alimentación, y se les facilitaba la transición suave de la UCIN al entorno del hogar.

Al final de la década de 1980, principios de 1990, se instauró la díada terapeuta-padres como un movimiento de intercambio colaborativo o sociedad. Se especificó que a medida que los conceptos de colaboración, comunicación y la potenciación se aplican, podían ser la dirección del futuro próximo. También, Schfsky ^57^, Cui *et al*. ^58^, Ross *et al*. ^59^, definen las pautas que requieren un terapeuta neonatal como parte del personal de las UCIN en un nivel de atención III o IV que incluye la atención de bebés en cualquier edad gestacional al nacer y con necesidades médicas y quirúrgicas complejas. Definen los roles de TO en la UCIN, y el uso de diferentes intervenciones terapéuticas realizadas con los lactantes de alto riesgo en la UCIN. Expresan que debido a la vulnerabilidad de los bebés prematuros, los terapeutas basados ​​en la UCIN necesitan habilidades avanzadas para optimizar los resultados del bebé, al mismo tiempo que comprenden y se adaptan a las intervenciones médicas que ocurren simultáneamente a las intervenciones terapéuticas

## Las herramientas para la intervención del terapeuta ocupacional facilitan la interpretación de su uso 

Hasta el momento se ha presentado la revisión acerca de los enfoques, métodos y teorías que utiliza el TO en la UCIN como herramientas de intervención, a partir de lo cual se comprende que el movimiento, la organización del tono muscular, la actividad y la postura respaldan la autorregulación del bebé; el esfuerzo del bebé para conseguir el equilibrio en las actividades de organización, así como la atención-interacción, manifiesta su capacidad para interactuar con el medio; las capacidades neurorregulatorias de cada niño guían las intervenciones dirigidas a optimizar los resultados del desarrollo neurológico ^20^^,^^28^.

El TO en neonatología, partícipe del grupo interdisciplinario ^17^^,^^18^, se guía, como ya se enunció, por los estándares determinados por la OMS ^1^, AOTA ^60^ los propuestos por Adrados ^61^ y Vergara *et al*^62^. Emplea enfoques específicos que fluyen de acuerdo al bebé como un ser singular que se ocupa, con uno factores físicos, cognitivos y afectivos individuales, manifiestos en una gama de habilidades, que consistentemente interactúan con el ambiente temporal, social, cultural y físico, en el cual se desarrolla el bebé, factores descritos en la Tabla 3.

**Tabla 3:** Factores interrelacionados en el desempeño ocupacional del neonato.

CCD	Ambiente inmediato
	Momento, secuencia y adaptación en el cuidado
	Ayudas para autorregulación en reposos
	Ayudas para autorregulación en cuidado
	Experiencias sensoriales apropiadas de acuerdo al desarrollo
CCF	Ambiente de la unidad
	Comunicación
	Participación en el cuidado
Neurodesarrollo	Proporciona experiencias sensorio-motoras
	Promueve el desarrollo de movimiento normal
	Desarrollo de patrones posturales

Fuente Vergara *et al*^20^

De acuerdo a lo representado, el TO reconoce al bebé como un ser ocupacional, que realiza ocupaciones, es copartícipe activo de patrones de acción que emergen de las interacciones del niño con el entorno. Kathleen ^63^, considera a las ocupaciones como “*las tareas y actividades que son valoradas, ya sea en la cultura de la familia o en la cultura de la UCIN, y en las que se espera que el bebé participe”;* “*las ocupaciones son patrones de acciones que emergen a través de interacciones entre el niño y el entorno”*. Así, se puede establecer que el desempeño ocupacional es la participación del bebé en tareas y actividades esperadas, ya sea por la familia o por el personal de la UCIN, en las que hay una ida y vuelta sincrónica entre las experiencias co-ocupacionales del bebé y los cuidadores. Este pensamiento induce al terapeuta ocupacional a acoplar la naturaleza del neonato con adaptaciones y modificaciones en el bebé, el cuidador en el medio ambiente o los objetos dentro de la UCIN ^64^^-^^66^. 

Entonces, el TO utiliza una relación transaccional entre el neonato, sus valiosas ocupaciones y su contexto, y maneja terapéuticamente las actividades de la vida cotidiana puesto que observa el estado de organización con los que interactúa, como explica Hunter ^23^; potencia o facilita la participación de los cuidadores en la creación de hábitos y rutinas, y las interacciones sensoriales, así como la modulación de las sensaciones, del espacio y los objetos como herramientas ^67^^,^^68^. 

Es por lo anterior que el papel del TO en los servicios de neonatología se direcciona a la intervención e investigación en la adquisición y la preservación de habilidades, de aquellos neonatos que tienen o están en riesgo de desarrollar una enfermedad, lesión, deficiencia, discapacidad, limitación de la actividad, o restricción de la participación incorporando a los familiares ^68^^-^^70^.

Al recapitular, en la revisión sistemática de artículos con miras a la actualización de contenidos y conceptos se encontró que las intervenciones del TO en neonatología no son normas ni recetas, son acciones individualizadas, específicas para cada niño, en cada momento. Parte de la observación de la respuesta del bebé, frente a los cambios o propuestas, corresponde ante todo preservar la estabilidad neuroconductual y fisiológica; hacer partícipe activo a la familia, al equipo de salud, esto con el propósito de influir en la calidad de vida presente y posterior del niño y su familia frente a situaciones que deben afrontar ^71^^-^^74^.

## Estrategias metodológicas y técnicas que utiliza la TO en su desempeño ocupacional 

En este espacio se hace una aproximación a que las estrategias y las técnicas de recolección o producción de datos están en función de la naturaleza de los prematuros. Desde observaciones para describir datos de los conversatorios o entrevistas para determinar un perfil ocupacional, se puntualizan los significados que le otorgan los profesionales de salud, las pautas estandarizadas para medir desempeño ocupacional del neonato y la familia, las técnicas que utilizan en sus intervenciones e investigaciones ^75^^,^^76^. 

Ya que muchos neonatos son examinados por profesionales que conocen sobre el desarrollo infantil, quienes están implicados en los distintos diagnósticos e intervenciones deben reconocer que además de los medicamentos existen otros acercamientos propuestos por otras disciplinas como el TO, quien propone al juego y lo lúdico como formas de estimular sensorialmente y promover las habilidades sociales, el control de movimientos y el desarrollo cognitivo; para tal fin es importante documentar la intervención y el impacto en la vida cotidiana del bebé y su familia ^71^^-^^76^.

Para desarrollar la práctica en la UCIN, el TO necesita conocer acerca de las condiciones, procedimientos médicos y vulnerabilidades de los recién nacidos. Además, reconocerá una comprensión de las habilidades de desarrollo individualizadas, las competencias de las teorías de organización neuroconductual neonatal ^19^^,^^20^, los sistemas de familia, los principios de desarrollo socio-emocional y el trabajo en equipo, entre otros. Solo de esta forma, el TO puede comprender cómo estos factores interactúan para influir en el desempeño del recién nacido. De acuerdo con lo expuesto, el pensar, hacer y ser del TO en neonatología abarca la prevención secundaria a través de la detección y del diagnóstico. Entre estos componentes de acción está conocer las creencias, prácticas culturales, necesidades en salud, oportunidades ocupacionales, sistemas políticos, ideologías e imaginarios que están detrás de las intervenciones médicas o clínicas ^76^. 

Durante la evaluación/intervención, el TO tiene presente la Organización neuroconductual, el desarrollo sensorial y el procesamiento sensorial ^77^. Por otro lado, considera la función motora referida al nivel postural, en la que se respeta el patrón flexor de piernas y brazos que se acercan al centro del cuerpo, dando la característica de posición fetal de manera simétrica o mecánica corporal. Utiliza los nidos y rollos para la comodidad, contención, estabilidad extrema, favorecer la postura flexora y evitar la deformidad craneal. Igualmente tiene en cuenta la actividad diaria (alimentación, vestirse, cambios de pañales, transferencias, estados de transición, dormir), así como el desarrollo socioemocional ^78^^-^^80^

En cuanto a la Familia, proceden los sistemas familiares, pensando en los estilos de aprendizaje para adultos; expectativas, esperanza de crianza cultural; interacciones entre padres e hijos, como el papel del padre durante la hospitalización, el cuidado informado y centrado en la familia y lo más importante, la transición del infante del hospital al hogar ^81^.

Un aspecto clave es el ambiente ^82^^-^^84^, 

*Ambiente sensorial* en el que se consideran cinco tipos:

- Táctil: sincronización, intensidad, textura, manejo para procedimientos, e interacción con los padres. Estimulación táctil propioceptiva que otorga un impulso facilitador de auto-organización
- Propioceptivo-vestibular: sincronización, intensidad, manejo para procedimientos e interacción con los padres (hamacas, balanceo del bebé sobre falda materna).
- Experiencias olfativas y gustativas específicas de la UCIN (tiempo, calidad, intensidad).
- Auditivo: Intensidad, duración, tiempo, animado versus inanimado.
- Visual: Temporización, ambiente e intensidad de la luz focal, contenido del campo *visual.*

*Ambiente social* en el que se identifica a padres e infantes; miembros de la familia ampliados y bebés; miembros del personal y bebé; padres y miembros del personal; terapeuta ocupacional, padres, personal e infante. 

*Ambiente físico* está conformado por los equipos y procedimientos médicos; la frecuencia, tiempo, duración, calidad e intensidad de la información sensorial de los médicos, equipo y procedimientos; la información sensorial de los equipos, procedimientos y actividades del personal que es perjudicial para la organización neuroconductual del bebé.

El *Ambiente cultural* se refiere a la filosofía de cuidado específica de la UCIN, incluida su particular orientación hacia cuidado agudo y crónico de bebés; los roles, funciones, actitudes y posiciones de los miembros del equipo en la organización estructural de la UCIN individual*;* la influencia de los factores estresantes de la UCIN; los patrones de comunicación y estructura, tanto formal como informal, entre los miembros del personal y entre la familia y el personal; reglas de comportamiento habladas y no dichas; el efecto del entorno físico y social en el rendimiento y la moral del personal, y la Humanización de la UCIN (incubadora con elementos personales, recuerdos familiares).

Siendo así, una parte integral del proceso de la terapia ocupacional es el uso terapéutico de uno mismo y este permite desarrollar y gestionar su relación terapéutica mediante el uso de la narrativa y el razonamiento clínico (empatía y un enfoque de colaboración). Mediante el uso de técnicas de comunicación interpersonal se desplaza el poder de la relación para que las familias tengan un mayor control en la toma de decisiones y la resolución de problemas, aspectos esenciales para una intervención eficaz ^83^^,^^84^. 

A modo de síntesis, para identificar las intervenciones de tratamiento utilizadas dentro de la UCIN por el TO, se tomó un ejemplo de Sardá, quien presentó algunas formas de realizar intervenciones con recién nacidos de alto riesgo, que pueden ser una guía para el acercamiento. En la Tabla 4 se enuncian las técnicas de disminución de la estimulación ambiental y estimulación sensorial unimodal ^85^.

**Tabla 4:** Las técnicas: Disminución de la estimulación ambiental. Estimulación sensorial unimodal

Reacciones de atención/interacción	
Frente a signos de estrés, suspender intervención. Un estímulo sensorial por vez. Luz tenue.	Mantener intervención ante signos de estrés. Interacción en condiciones inadecuadas. Más de 1 estímulo sensorial simultáneo.
Mantenerlo cubierto, envuelto o vestido. Estímulo visual a 30-40 cm. de la vista. Eliminar estímulos innecesarios. Facilitar actividad mano-boca o chupete. Postura en flexión. Dar sostén y estabilidad. Intervención RN en alerta.	

Fuente: Sardá ^85^^).^

## Conclusiones

Lo expuesto representa la visión general de los requerimientos para el acercamiento del TO en neonatología. 

Referente al desarrollo de los prematuros, se recapacita que si las necesidades individuales no se estimulan, a largo plazo son evidenciadas en afectación de: la capacidad de la praxis, la imitación, la secuenciación y la construcción; las habilidades motoras; las capacidades cognitivas, como la percepción; las habilidades de procesamiento (la capacidad de organizar las acciones de una manera oportuna y segura), y las capacidades de regulación emocional que pueden afectar la habilidad para responder eficazmente a las demandas de ocupación con una gama de emociones.

En consecuencia, más allá del modelo de referencia centrado en el problema convencional de la discapacidad, los TO hacen una valiosa contribución al cuidado de los RN ya que se centran en el poder del compromiso con la ocupación y el apoyo a los padres en el cuidado del bebé con el fin de lograr resultados positivos para la familia y los cuidadores.

Es un cambio de actitud de 

***“somos los expertos y sabemos que es lo mejor para el paciente”***

***a “nosotros lo hacemos contigo”*** Zarubi et al ^86^.

Si entendemos lo interdisciplinario como ese diálogo e intercambio de técnicas, conceptos y métodos investigativos entre diferentes disciplinas, la TO, sin perder su identidad, no tiene problemas en aprehender herramientas heurísticas de otros campos disciplinarios para el ejercicio de la investigación. La intervención de TO en la UCIN es una práctica colaborativa en la que al mismo tiempo se demuestra respeto y apoyo a las funciones de otras personas y las disciplinas en las vidas de los neonatos. 

Conocer la idiosincrasia de la estructura social, cultural, creencias, prácticas culturales, necesidades en salud, de familiares y profesionales de salud brinda oportunidades ocupacionales, pertinentes a las intervenciones del TO, que están detrás de las intervenciones médicas o clínicas.

Compartir nuestras experiencias, aciertos y errores, dificultades y equivocaciones, generar y validar instrumentos, pautas estandarizadas e intervenciones clínicas, medir su eficacia e identificar situaciones por corregir nos presenta como “investigadores activos” que enseñan no solo lo que se ha hecho, sino lo que se está haciendo y lo que puede cambiar. Consejos y orientaciones con base en experiencias reales de investigación pueden contribuir al “cómo” enfrentarse al proceso de investigación a partir de la documentación de las observaciones, las intervenciones, su impacto para difundir y comunicar públicamente; de esa forma vamos construyendo historia y prácticas del TO en neonatología, es un símbolo de plantear el carácter práctico y transformador de la praxis ocupacional.
